# Pleural Schwannoma Presenting As Musculoskeletal Type Pain: A Case Report

**DOI:** 10.7759/cureus.37771

**Published:** 2023-04-18

**Authors:** Ali El-Husari, Hozaifa Tabbaa, Mohamed Ibrahim, Davong D Phrathep, Abubakr Bajwa

**Affiliations:** 1 Osteopathic Medicine, Lake Erie College of Osteopathic Medicine, Bradenton, USA; 2 Pulmonology and Critical Care, Ascension Medical Group St. Vincent's Lung Institute, Jacksonville, USA

**Keywords:** rare benign tumor, s100, intermittent chest pain, benign peripheral nerve sheath tumor, pleural schwannoma

## Abstract

Schwannomas are benign peripheral nerve sheath tumors typically found in the neck, flexor surfaces of the extremities, mediastinum, posterior spinal roots, cerebellopontine angle, and retroperitoneum. Pleural schwannomas are a type of neoplasm that arises from autonomic nerve fiber sheaths in the pleura and rarely originate in the thoracic cavity. These schwannomas tend to be asymptomatic, benign, and slow-growing neoplasms. Although pleural schwannomas commonly occur in males, our report highlights a unique presentation of a pleural schwannoma presenting as musculoskeletal-type chest pain in an adult female. Our patient's diagnosis of pleural schwannoma was supported after X-Ray, Computed Tomography (CT) Scan, and Positron Emission Tomography (PET) Scan imaging was complete. All imagining and immunohistochemical staining yielded pleural schwannoma as the final diagnosis. We aim to bring awareness to the necessity of imaging and histopathological staining in atypical clinical cases of pleural schwannoma. Our novel case highlights pleural schwannoma as a differential diagnosis for patients with intermittent, musculoskeletal-type chest pain.

## Introduction

Schwannomas are rare tumors from Schwann cells. They commonly arise within the skull base, cerebellopontine angle, and posterior spinal roots [1]. However, they can also be found in the neck, flexor surfaces of the extremities, mediastinum, and retroperitoneum [2]. Primary pleural schwannomas are rare, comprising only 1-2% of all thoracic tumors [1]. They arise from autonomic nerve fiber sheaths in the pleural surface of the lung [3]. They are commonly found in adults in the third to a fourth decade, with increased incidence in males [3]. Diagnosis of pleural schwannoma is usually incidental as they are often solitary, slow-growing, benign, asymptomatic tumors [4]. Here we report a case of primary pleural schwannoma in a 33-year-old female, found on a chest CT scan after reporting months of intermittent left-sided musculoskeletal type chest pain.

## Case presentation

A 33-year-old female presented to her primary care complaining of intermittent left musculoskeletal-type chest pain she had been having for a few months. She was sent to radiology, where she underwent an X-ray and computed tomography (CT) imaging. This revealed a left-sided pleural-based nodule in the lateral mid-thorax area adjacent to her third rib. The nodule was smooth-edged and measured 11x19mm. The patient was referred to pulmonology. 

On clinical assessment, she reported no weight loss, fever, cough, or other symptoms besides intermittent pain, which she rated 3/10. Her physical exam was significant for tenderness on palpation of her left upper rib cage. She had no smoking history or history of exposure to chemicals or radiation, and her family history was positive for lung cancer in more than 1 family member. Her symptoms and imaging raised suspicion of sarcoma of sorts. Therefore, Positron Emission Tomography (PET) scan and bronchoscopy with a CT-guided core biopsy were ordered.

**Figure 1:**
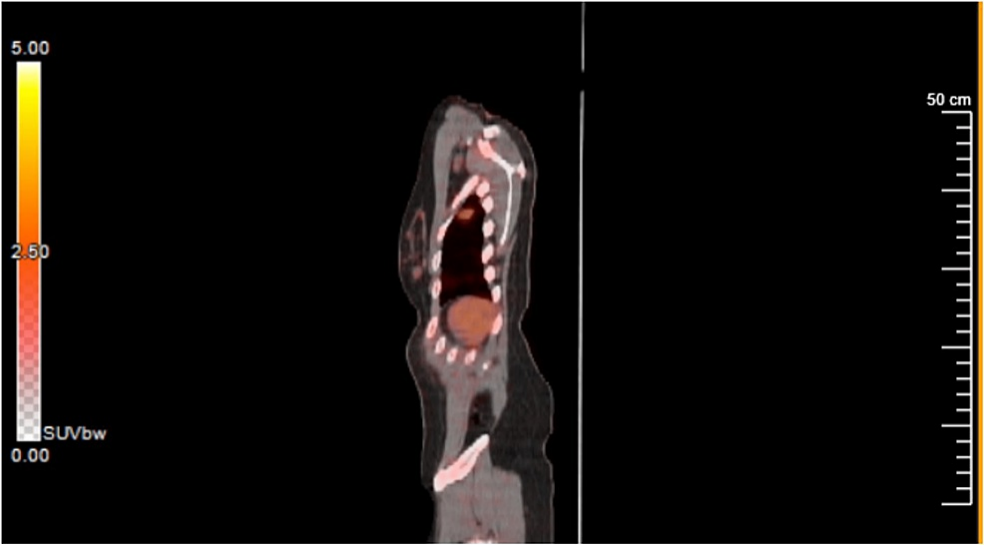
A subpleural pulmonary nodule is seen in the left upper lobe with an SUV of 2.13. Physiological uptake is also seen within the abdomen.

**Figure 2:**
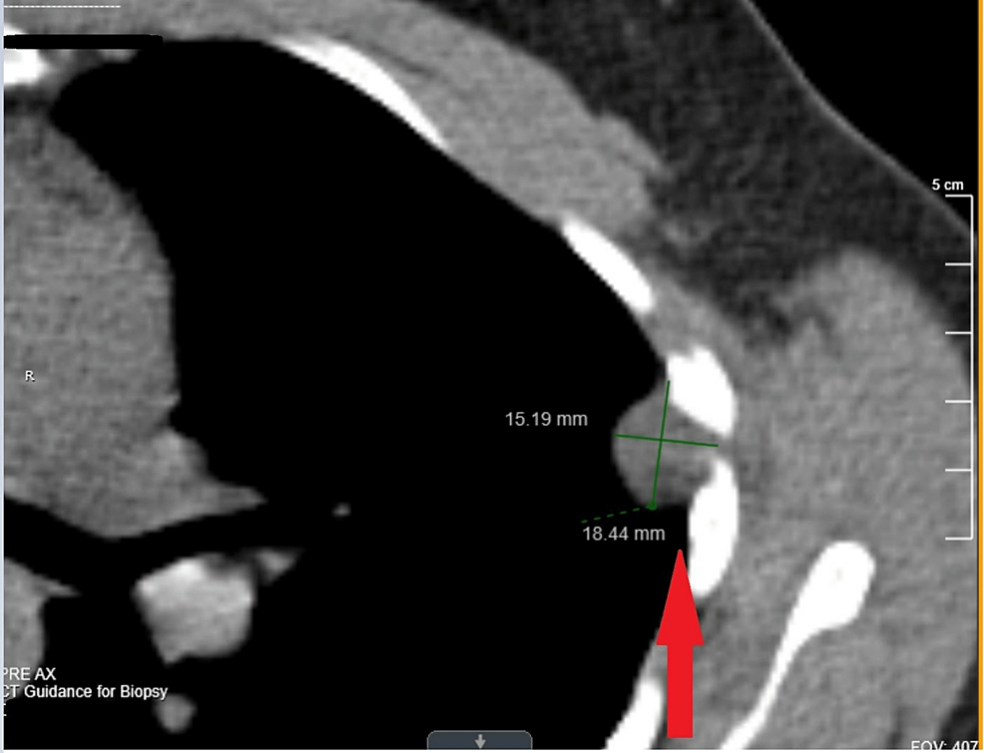
The nodule is seen adjacent to the ribs. The nodule measures roughly 15.19x18.44mm in this view.

The patient's PET scan (Figure 1) showed mild Fluorodeoxyglucose (FDG) uptake with a maximum standardized uptake value (SUV) of 2.13. This decreased suspicion of malignancy as malignant levels of SUV are usually 2.5 or greater. The patient underwent bronchoscopy with a CT-guided core biopsy, which yielded 15 samples. The CT scan is taken before the bronchoscopy, as seen in Figure 2. The surface of the nodule was soft but solid with a gray gelatinous appearance. The inside of the sample was a more pale, tan tissue. Pathologic evaluation revealed spindle cells with no definite mitotic activity or marked nuclear polymorphisms. Immunohistochemical staining was positive for saturated ammonium sulfate (S100), SRY-Box Transcription Factor 10 (SOX 10), Wilms Tumor 1 (WT 1), Cluster of Differentiation 99 (CD99), and B-Cell Lymphoma 2 (BCL-2). It was negative for Signal transducer and activator of transcription 6 (STAT 6), c-kit (CD17), calretinin, pan-cytokeratin, Cluster of Differentiation 31 (CD31), survival motor neuron (SMA), Multiple Sequence Alignment (MSA), desmin, Mclan-A, and Activated Fibrin Stabilizing Factor (FXIIIa). These results revealed the mass to be a peripheral nerve sheath tumor or schwannoma. The patient was referred to surgery.

**Figure 3:**
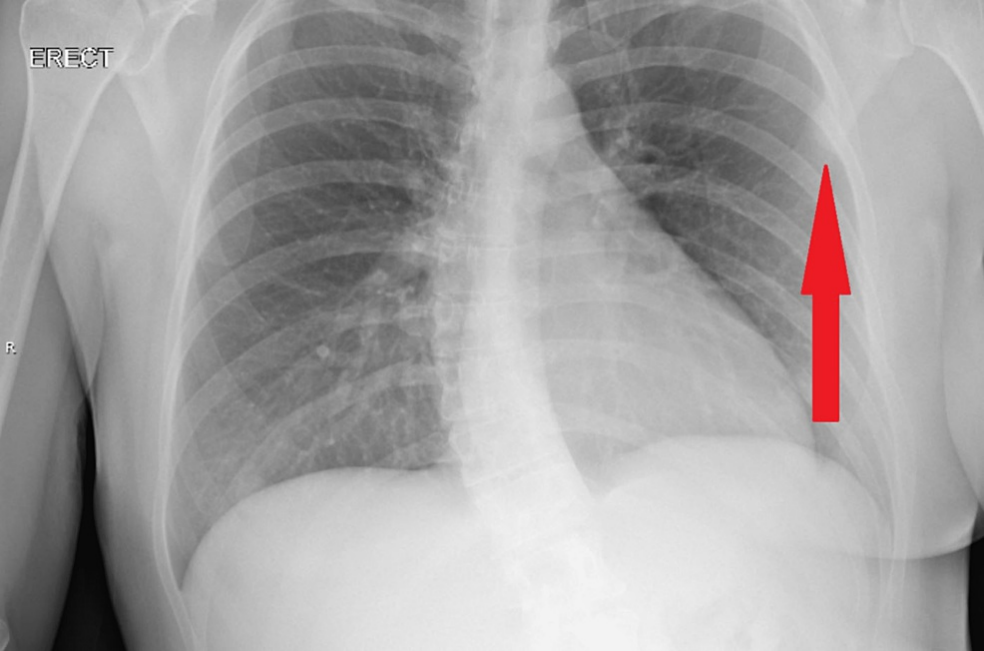
Anterior posterior radiograph pre-surgery show normal cardio-mediastinal silhouette. The pleural based nodule is seen above the arrow, in the left upper lobe adjacent to the third rib. There is no edema or airspace consolidation. Scoliosis noted in thoracolumbar spine.

**Figure 4:**
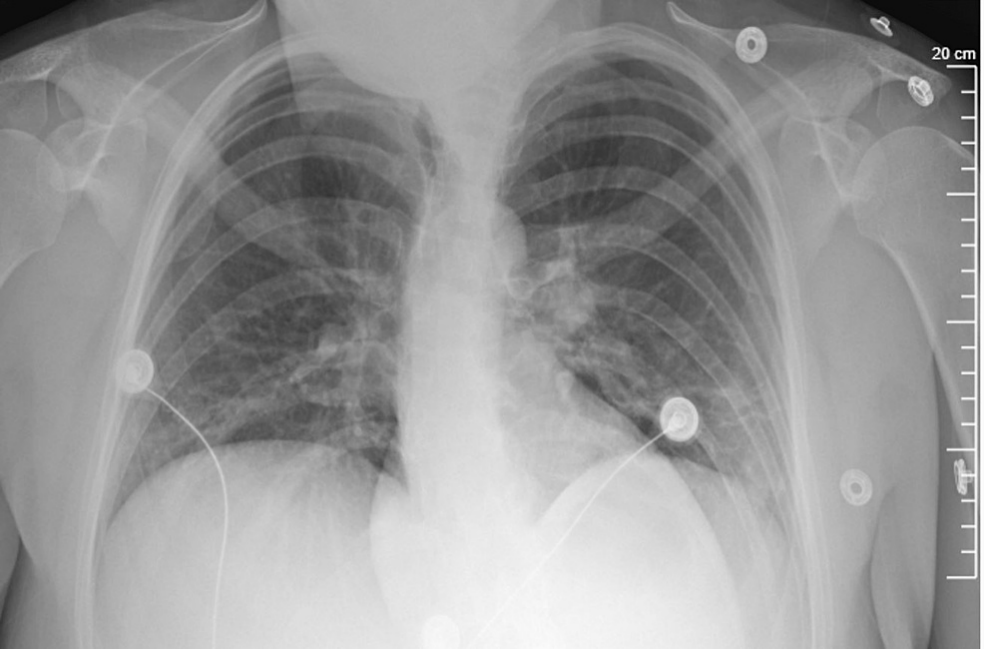
Anterior posterior radiograph of patient post operation. Nodule was removed in its entirety.

The patient underwent robotic wedge resection via thoracotomy. The laterally placed chest wall schwannoma was seen protruding into the pleural space. The entire lesion was excised with some intercostal muscle margins in toto. X-ray images pre and post-surgery are seen in Figures 3-4, respectively. The histopathological evaluation revealed a benign tumor with healthy margins. The cut surface of the nodule was described as soft but solid, gray, and gelatinous. Further evaluations confirmed the mass to be a schwannoma with minimal attached soft tissue.

The patient recovered well from surgery with no complications. She was seen on follow-up a month later. She reported no recurrence of her original pain, had no further symptoms, and was cleared to be seen as she needed in the future.

## Discussion

Pleural schwannomas are one classification of peripheral nerve sheath tumors (PNST) that are benign neoplasms rarely found in the thoracic cavity and even less common in the pulmonary pleura. PNSTs are usually located in the posterior mediastinum and are rarely found in other mediastinal compartments [5]. A study of 75 intrathoracic PNSTs found that only 21 were benign pleuropulmonary PNSTs, while 49 were benign mediastinal PNSTs [5]. Of the 21 benign pleuropulmonary PNSTs, 13 were schwannomas, while the others consisted of neurofibromas, peri-neuromas, and ganglioneuromas [5]. While the latter three types tend to be of endobronchial origin, schwannomas mostly occupy the pleural space where innervation is by the intercostal nerves [5]. 

Though other PSNTs can initially present with symptoms of pain, palpable/visible mass, or nerve palsy [6], pleural schwannomas have largely been known to be asymptomatic. Some other cases have reported symptoms such as shortness of breath, cough, hemoptysis, and pleural effusion [1-4]. In our case, the patient presented with musculoskeletal-type pain only, which was not seen in previous cases. 

There are several possible explanations for the pain. First, the interaction of the pleural schwannoma with intercostal nerves causes the patient to have pain. Sughrue et al. [6] explained a few other possible mechanisms. They first theorized that the pain could be explained by the Schwann cell lineage's role in producing neuropathic pain. In response to injuries, Schwann cells produce cytokines involved in producing neuropathic pain. Another theory explains how Schwann cells play a role in guiding sprouting axons during neuronal regeneration in post-traumatic injuries. A lack of Schwann cells causes neurons to sprout in random directions. It does not allow for proper neuronal connections, which have been hypothesized to lead to random neuron firing, causing neuropathic pain. 

Diagnosing pleural schwannomas is challenging, and radiological imaging can produce ambiguous results. Nonetheless, CT imaging showing solid, solitary, and well-defined pleural tumors should prompt adding schwannoma into the differential diagnosis [7]. The definitive diagnosis, however, still requires histopathological studies and immunohistochemistry staining of a tissue sample. In our case, histopathologic evaluation of the biopsies showed positive immunohistochemistry staining for S100 and SOX 10, two markers with the highest sensitivity and specificity for schwannomas [8].

A case report published in 2014 illustrated that less than 20 cases had been reported in the medical literature [3]. However, there have been at least five case reports on pleural schwannoma in the last five years. This could be due to increased surveillance via CT; however, each has also presented with different symptoms. Due to the increase in the number of pleural schwannomas recorded in recent years with no consistent symptoms, we urge doctors to consider pleural schwannomas in their differential list as they have a good prognosis if discovered and treated early.

## Conclusions

Schwannomas are a rare type of peripheral nerve sheath tumor. While most reside in the posterior aspects of the human body, such as the posterior mediastinum and retroperitoneum, pleural schwannomas have begun to be reported due to the increased collection of nerves in those regions as coincidental findings on radiologic imaging. The presentation of most pleural schwannomas is asymptomatic, making pinpointing this disease difficult through symptoms alone. Our patient presented with a few months of intermittent left-sided musculoskeletal-type pain that compelled her to present to a pulmonologist for evaluation. The peripheral pleural mass was identified and excised. To further identify this tumor accurately, physicians are encouraged to focus more on pulmonary and mediastinal sources of pain when a patient presents with chest pain, as many are trained and used to emphasizing cardiac causes. If recognized early, the treatment options and prognosis are excellent, prompting us as a healthcare community to evaluate all causes of chronic chest pain with an open mind and consider all options.
